# Enhancement in dopamine reduces generous behaviour in women

**DOI:** 10.1371/journal.pone.0226893

**Published:** 2019-12-31

**Authors:** Sergio Oroz Artigas, Lu Liu, Sabrina Strang, Caroline Burrasch, Astrid Hermsteiner, Thomas F. Münte, Soyoung Q. Park

**Affiliations:** 1 Department of Psychology, University of Lübeck, Lübeck, Germany; 2 Department of Decision Neuroscience & Nutrition, German Institute of Human Nutrition, Nuthetal, Germany; 3 Deutsches Zentrum für Diabetes, Neuherberg, Germany; 4 Department of Neurology, Universitätsklinikum Schleswig-Holstein, Lübeck, Germany; 5 Neuroscience Research Center, Charité-Universitätsmedizin Berlin, Corporate Member of Freie Universität Berlin, Humboldt-Universität zu Berlin, and Berlin Institute of Health, Berlin, Germany; Daegu University, REPUBLIC OF KOREA

## Abstract

Generosity is a human behavior common in social contexts. However, humans are not equally generous to everyone alike. Instead, generosity decreases as a function of social distance, an effect called social discounting. Studies show that such social discounting effect depends on diverse factors including personality traits, cultures, stress or hormonal levels. Recently, the importance of the neurotransmitter dopamine in regulating social interactions has been highlighted. However, it remains unclear how exactly dopamine agonist administration modulates generous behavior as a function of social discounting. Here, we investigate the causal effect of dopamine agonist administration on social discounting in a pharmacological intervention study. We employ a randomized, double-blind, within-subject design to investigate the impact of the D2/D3 receptor agonist pramipexole on social discounting by keeping gender constant. We apply hyperbolic social discount model to the data and provide evidence that women under pramipexole become less generous in general, especially towards close others. Our results highlight the crucial role of dopamine in social decision making.

## Introduction

Generosity is a common human behavior defined as the willingness to benefit others without expecting anything in return [1,2]. Specifically, it has been shown that generosity decreases with increasing social distance, an effect referred to as social discounting [3]. In recent research, this phenomenon has been shown to be modulated by diverse factors. For instance, previous studies demonstrate that individual differences in personality measures, such as empathy [4] and cognitive load, change social discounting [5]. Further, cultural differences were shown to modulate generous behavior as a function of social discounting [6]. Interestingly, different biological factors also play a crucial role in modulating social discounting. Intranasal oxytocin interacts with individual differences in empathy on the social discounting task [4]. In line with this, stress shapes social discounting behavior [7], whereas cortisol and noradrenaline have dissociable roles in social discounting. While cortisol promotes prosocial behavior towards close individuals, noradrenaline has an opposing effect, as it inhibits generosity [8].

On the neural level, neuroimaging studies delivered evidence in how social discounting involves brain regions, such as the orbitofrontal cortex (OFC) which is influenced by the neurotransmitter dopamine [3]. Dopamine has been suggested to play a pivotal role in decision mechanisms, both in social and non-social contexts. Investigations in the last decades emphasize its role in reward processing [9], as well as its involvement in coding reward both towards self and towards others [10]. Furthermore, dopamine modulates impulsivity as shown in risk taking decisions [11] and temporal discounting behavior [12]. Influence of dopamine levels extends to the social contexts. For instance, higher levels of dopamine correlate with social status [13] and promote egalitarian behavior [10]. In line with this, Soutschek and colleagues [14] showed that administration of the dopamine antagonist amisulpride increased generosity in men, but decreased it in women. According to a theoretical assumption in gender differences in social preferences [15], women prefer more prosocial- than self-reward, and vice versa for men. Thus, dopamine receptor blockade may reduce the reward biases in both women and men in opposite directions. However, it is still unknown how the dopamine agonist impacts the individual social discounting.

The goal of the present study is to elucidate the impact of pramipexole, a D2/D3 receptor agonist (DA), on social discounting, by using a randomized, double-blind, within-subject design. Based on the previous work, showing gender differences in dopamine driven social discounting modulation, we kept the gender constant by investigating female participants. Subjects came on two different days with a gap of at least 5 days in between. In each experimental session, participants performed a social discounting task after oral administration of either pramipexole (DA) or placebo (PLC). Sharing behavior was modeled using a hyperbolic discounting function.

## Methods

### Subjects

Thirty-three healthy women (22.24 ± 2.53, mean age ± SD) were recruited for the experiment. Exclusion criteria were age below 18 or above 35, BMI below 18 or above 25, food allergies or other dietary constraints, drug consumption, alcohol consumption above 15 cups per week, any history of mental disorder or medical disease and current medication (except for birth control). All subjects gave written informed consent after being instructed about the procedure according to the Declaration of Helsinki and they were informed that they would receive a payment of 8€ per hour. The study was approved by the local Ethics Committee of the University of Lübeck.

### Social environment measurement

Directly after the DA or placebo administration while waiting for the drug to take effect, participants were asked to describe their social environment [3]. Their social environment was represented by a horizontal scale consisting of 101 avatars. The leftmost avatar represented the subject herself and the avatar closest to the self was labeled as social distance 1, representing the person socially closest to them (for example their mother, father or boyfriend). The rightmost person was labeled as social distance 100 and represented a stranger, while social distance 50 indicated a person they might have seen, but whose name they do not know. Subjects were asked to indicate names of representatives for social distances 1, 2, 3, 5, 10, and 20 of their own social environment [4]. Importantly, they were instructed to only include people they have positive feelings for.

### Social discounting task

Social discounting was assessed by a computerized dictator game in combination with the social distance scale as described above. In each trial subjects received an endowment (€5, €7 or €8) and they had unlimited time to decide how much of it they want to share with a person at a given social distance (1, 2, 3, 5, 10, 20, 50 or 100). Participants performed the task on a computer and played a total of 24 randomized trials. At the end of the experiment, one trial was randomly selected and subjects were paid according to the split of that trial. Further, the receiver in the chosen trial received the money subjects decided to share with them. Before the experiment, participants were informed about the payment procedure and were asked to indicate contact details of the corresponding person after the experiment, for later money transfer. The dependent variable was the averaged percentage of money shared within each social distance.

### Procedure

Subjects were tested at two days separated by at least 5 days. One day they received a capsule containing mannitol-siliciumdioxid (PLC condition) and the other day a visually identical capsule with 0.35mg pramipexole (a D2/D3 receptor agonist; DA condition; SIFROL®, Zentiva GmbH, Frankfurt/Main, Germany). We used a double-blind design and the order of placebo and drug was counterbalanced across subjects. On both days, they additionally received 1ml domperidone (= 10 mg; Motilium®, Takeda GmbH, Konstanz, Germany) in order to prevent potential nausea, a frequent side effect of pramipexole.

Since nutrition could have an impact on dopaminergic function itself [16], subjects were asked to refrain from eating and consuming caloric beverages starting at 8.00 pm on the day before the two experimental sessions. On both days, participants arrived at the laboratory either at 7.30 or at 8.30 am. 30min after arrival, subjects received pramipexole or placebo together with domperidone. Three hours after the pramipexole/placebo intake, subjects were asked to perform the social discounting task which corresponds to the pharmacokinetic properties of the pramipexole reaching a maximum concentration after 1–3 hrs [17]. Subsequently subjects were asked to stay in the lab up to three more hours to monitor and treat potential drug side-effects. At the end of each session subjects were asked whether they thought they had received the drug or the placebo. Awareness of drug condition was tested with a binomial test.

### Data analysis

A standard hyperbolic function was applied to quantify the degree of social discounting (Eq 1):

$$sv=\frac{V}{1+k*(D-1)}$$ (1)

Here s𝑣 represents the discounted value of the reward that the other person regarding (the percentage of money shared). V is the undiscounted value of this reward and can be interpreted as a reference-point for the money to share, representing individual’s overall level of generosity. D represents the social distance and k is a constant describing the degree of discounting. Larger k corresponds to a steeper decrease in generosity as social distance increases. Individual social-distance-dependent changes in generosity were estimated by fitting this hyperbolic function to the percentage of money shared at each social distance on an individual level per condition. The parameter V was used to estimate subjects’ overall levels/reference-point of generosity independent of social distance, with larger values indicating higher generosity especially to close others. The parameter k represents the general degree of social discounting, that is the decline of generosity with increasing social distance, with higher values indicating a steeper decline. In order to evaluate the goodness-of-fit of the function to the data, R-squared was used on the group level to evaluate the goodness-of-fit of the model to the data.

We performed t-tests for dependent samples to test for differences in the k and V parameters between the DA and PLC condition. In order to test whether DA exerts a general effect on generosity, independent of social distance, the area under the curve (AUC) of the shared money of each subject across social distances was compared between the conditions by employing a t-test for dependent samples.

The data of three participants were excluded from analysis: two of them did not complete the second session, while a third participant showed the same behavior throughout the entire experiment (sharing 100% of the endowment), we could therefore not ensure whether she understood the instruction. As a result, the data of remaining 30 subjects were included for analyses.

## Results

### Subjects are unable to distinguish treatment and placebo

Before testing the impact of DA on social distance, we checked the participants’ guess of drug intake. 47% them were correct, with the binomial test indicating that this percentage does not significantly differ from chance level (50%; p = 0.856).

### Impact of pramipexole on k and V parameters

**Fig 1:**
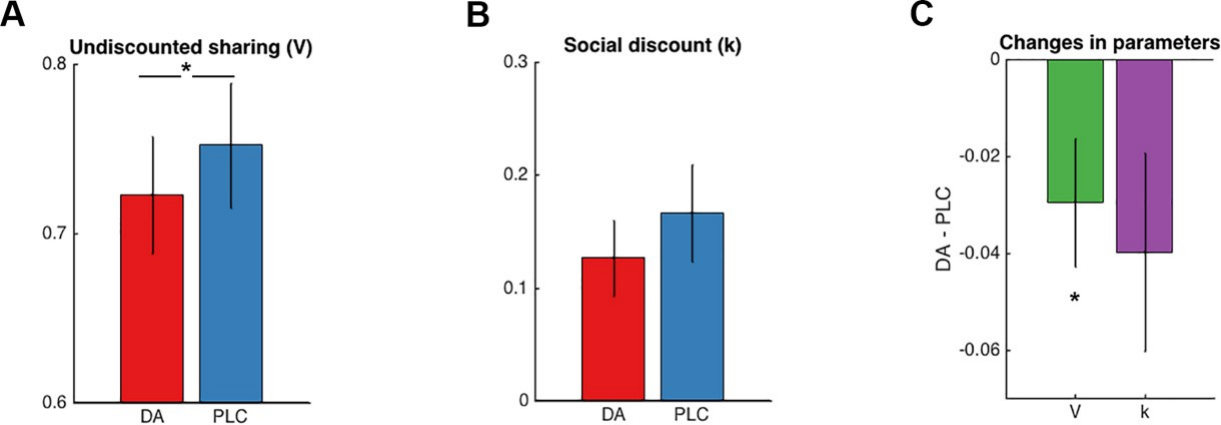
DA receptor agonist pramipexole reduces generosity reference-point (V). Comparisons between conditions show that parameter V representing the overall generosity levels especially to close others, is lower under DA than PLC (t(29) = -2.225, p = 0.034) (A). We observe that parameter k representing social discount showed a difference in trend (t(29) = -1.949, p = 0.061) (B). The **condition-**comparison between DA and PLC in parameter V and k (C). Error bars represent SEM. *p < 0.05.

We tested whether DA had an impact on social discounting by comparing the k and V parameters between conditions. Estimated V parameters were significantly lower under DA than PLC (t(29) = -2.225, Cohen’s d = -0.397, p = 0.034* (Fig 1A and 1C). This means less sharing behavior with close relatives under DA than under PLC. We also observed a difference of k parameter in trend, suggesting that subjects under PLC showed steeper decreases in social discounting than under DA (t(29) = -1.949, Cohen’s d = -0.413 p = 0.061 (Fig 1B and 1C). Importantly, this effect cannot be explained by a general, social distance independent, effect of DA on generosity since the AUC did not differ between conditions (t(29) = 0.379, p = 0.707).

The hyperbolic model provided a good fit of the data on the group level as measured by R-squared for both DA (R^2^ = 0.751, SE = 0.035) and PLC (R^2^ = 0.769, SE = 0.038). Goodness-of-fit did not differ between treatments (t(29) = -0.491, p = 0.627).

A robustness check was conducted by using maximum-likelihood estimation. We found quite similar results (Table 1) which further supported the findings using least squares estimation.

**Table 1:** Results of robustness check.

Variables	DA	PLC	*t*	*p*
MLE				
Parameter k	0.1264	0.1664	-1.931	0.0633
Parameter V	0.7227	0.7523	-2.226	0.0339
*R*^*2*^	0.7505	0.7687		
LSE				
Parameter k	0.1265	0.1663	-1.949	0.0611
Parameter V	0.7227	0.7522	-2.225	0.034
*R*^*2*^	0.7506	0.7688		

MLE, maximum-likelihood estimation; LSE, least squares estimation; DA, administration of dopamine receptor agonist pramipexole; PLC, administration of placebo.

## Discussion

Our results provide evidence that D2/D3 receptor agonist pramipexole impacts social discounting in women. Based on the individual parameters from the hyperbolic discounting model, we show that parameter V decreases under DA compared to placebo, indicating that as a general generosity reference point, participant’s undiscounted value is reduced under the influence of pramipexole. Furthermore, we observe a trend towards a reduction of the k parameter under pramipexole, suggesting that female subjects with DA enhancement tend to discount less as a function of social distance. However, this effect depending on the social discounting and pramipexole did not exert a general influence on generous behavior, independent of social distance. We did not observe any significant differences in the AUC of the discounting function. Importantly, these effects were present without participants’ awareness of the drug condition.

Our results show a selective change of V parameter under pramipexole. One explanation is that DA may enhance self-reward. In line with this, a previous study using L-Dopa in combination with an economic bargaining game has shown that an enhancement in the dopaminergic system may drive greater self-reward in the absence of punishment threat, whereas with the punishment threat, this effect disappears [18]. According to this study, dopamine enhancement tackles decision-making mechanisms, rather than simply enhancing reward representation. Moreover, the sharing behavior towards distant others was markedly reduced with a trend in increase after pramipexole treatment. Previous studies have demonstrated the in-group out-group effects, of which the intranasal oxytocin promoted in-group cohesion, cooperation and norm enforcement [19,20]. In line with this, a previous study using the social discounting task has confirmed the enhancement of oxytocin in sharing behavior to close others [4]. Opposite to the OT-effect in in-group social behaviors, dopamine may increase egalitarian tendencies (inequity-averse behavior) in reward and decrease the hyperaltruism in punishment to strangers [21,22]. In other words, DA may reduce the disparity/discrimination between self, close-others and strangers, thereby reducing the social discounting. In line with this, our results show that the DA agonist pramipexole reduced the sharing behavior to close others, while it decreased in trend the social discounting in general.

Our results, showing how a dopamine agonist reduces overall generosity levels especially to close social distances, can draw novel hypotheses about its underlying neural mechanisms. Current neural models of generosity suggest that self-reward is coded in the ventral-medial prefrontal cortex (vmPFC) and is inhibited by activity in temporal-parietal junction (TPJ) during generous decisions [3]. In line with this model, DA may selectively change vmPFC activation [23], with dopaminergic projections overcoming TPJ inhibition and thus promoting selfish decisions. This idea is supported by a previous study investigating the effect of a single-dose pramipexole in Parkinson’s disease in the framework of delay discounting decisions combined with Positron Emission Tomography [24]. In this study, patients reveal an augmentation in impulsive choices after pramipexole submission, accompanied by a change in medial prefrontal cortex [24].

Furthermore, according to the theoretical assumption in gender differences in social preferences [15], women in general prefer more prosocial- than self-reward, and vice versa for men. Thus, in our study dopamine receptor agonist may reduce the reward biases in women via enhancing the weight of self-reward, especially in the self versus close-others situation. Interestingly, a recent study has investigated social discounting by applying a D2-receptor antagonist amisulpride, which indicated the gender differences in the treatment effect in prosocial sharing behavior [14]. Specifically, female participants became more selfish, whereas male participants became more generous when sharing with close others under amisulpride. This effect was visible by the significant changes in V parameter. The results may indicate the amisulpride’s blockage effect via inhibiting the dominant prosocial-reward in females (vice versa for males). In addition, amisulpride did not change k parameter at all, whereas in our data, the k parameter was in trend reduced with the intake of pramipexole. That is, pramipexole induced a trend in generosity modulation as a function of social distances in females. Taken together, both dopamine agonist and antagonist in females lead to a reduction in generosity-reference point. To fully understand the exact role of dopamine in different genders, future studies are required investigating the mechanisms of dopamine agonists versus antagonists in decision-making and interpersonal behavior across genders in a within-subject design.

Chronic administration of pramipexole applied for the treatment of PD patients, showed positive effects on reducing motor symptoms [25], and some presented effects on higher cognitive functions like working memory [26] and generosity [27]. Although these effects might be due to chronic administration in patients, experiments on healthy subjects receiving acute doses of pramipexole also showed alterations on reward processing [23], providing cumulative evidence for the effectiveness of pramipexole as a DA receptor agonist on high cognitive functions. Nevertheless, further studies are needed to verify the mechanisms and effectiveness of pramipexole oral administration on complex cognitive function, such as decision making [28].

Taken together, we show that female participants become less generous toward socially close individuals under the influence of a dopamine agonist pramipexole, whereas they, to some extent, become more egalitarian in general toward others across social distances. Our results contribute significantly in understanding the exact role of dopamine on social decision making.

Our study has several limitations. First, increasing evidence indicated the impact of estrogen on dopamine-dependent cognitive processes (e.g., working memory, reward processing and inhibitory control) [29,30], which implicates that menstrual cycle may impact social sharing behaviors by interacting with the dopaminergic systems. Thus, the menstrual cycle and estrogen need to be assessed in future studies to further revealing its possible effects via dopamine activation. Second, in this study, we investigated only females to control for possible gender confounds in DA effects in social generosity. Gender differences has been reported by a previous study investigating social discounting with dopamine antagonist [14]. This, of course constrained the generalizability of our results. However, it is important to mention that the gender comparison was not main focus of this study. It is noteworthy that the investigation of both males and females would be required in future. Furthermore, studies with small sample size have been demonstrated to have larger effect sizes than larger ones, that is the inflated effect size, which further hampers the replication process [31,32]. Thus, the findings of our study remain to be replicated in future studies with larger sample size.

## References

[1] Park SQ, Kahnt T, Dogan A, Strang S, Fehr E, Tobler PN. A neural link between generosity and happiness. Nature Communications. 2017 7 11 10.1038/ncomms15964 28696410 PMC5508200

[2] Strang S, Park SQ. Human Cooperation and Its Underlying Mechanisms (pp. 223–239). Springer, Cham; 2016 10.1007/7854_2016_445. 27356520

[3] Strombach T, Weber B, Hangebrauk Z, Kenning P, Karipidis II, Tobler PN, et al Social discounting involves modulation of neural value signals by temporoparietal junction. Proceedings of the National Academy of Sciences of the United States of America. 2015; 112: 1619–1624. 10.1073/pnas.1414715112 25605887 PMC4321268

[4] Strang S, Gerhardt H, Marsh N, Oroz Artigas S, Hu Y, Hurlemann R, et al A matter of distance-The effect of oxytocin on social discounting is empathy-dependent. Psychoneuroendocrinology. 2017; 78: 229–232. 10.1016/j.psyneuen.2017.01.031 28219815

[5] Strombach T, Margittai Z, Gorczyca B, Kalenscher T. Gender-Specific Effects of Cognitive Load on Social Discounting. PloS One. 2016; 11: e0165289 10.1371/journal.pone.0165289 27788192 PMC5082848

[6] Strombach T, Jin J, Weber B, Kenning P, Shen Q, Ma Q, et al Charity Begins at Home: Cultural Differences in Social Discounting and Generosity. Journal of Behavioral Decision Making. 2014; 27: 235–245. 10.1002/bdm.1802

[7] Margittai Z, Strombach T, Van Wingerden M, Joëls M, Schwabe L, Kalenscher T. A friend in need: Time-dependent effects of stress on social discounting in men. Hormones and Behavior. 2015; 73: 75–82. 10.1016/j.yhbeh.2015.05.019 26122295

[8] Margittai Z, van Wingerden M, Schnitzler A, Joëls M, Kalenscher T. Dissociable roles of glucocorticoid and noradrenergic activation on social discounting. Psychoneuroendocrinology. 2018; 90: 22–28. 10.1016/j.psyneuen.2018.01.015 29407513

[9] Schultz W. Multiple Dopamine Functions at Different Time Courses. Annual Review of Neuroscience. 2007; 30: 259–288. 10.1146/annurev.neuro.28.061604.135722 17600522

[10] Sáez I, Zhu L, Set E, Kayser A, Hsu M. Dopamine modulates egalitarian behavior in humans. Current Biology. 2015; 25: 912–919. 10.1016/j.cub.2015.01.071 25802148 PMC4627633

[11] Burke CJ, Soutschek A, Weber S, Raja Beharelle A, Fehr E, Haker H, et al Dopamine Receptor-Specific Contributions to the Computation of Value. Neuropsychopharmacology. 2018; 43: 1415–1424. 10.1038/npp.2017.302 29251282 PMC5916370

[12] Joutsa J, Voon V, Johansson J, Niemelä S, Bergman J, Kaasinen V. Dopaminergic function and intertemporal choice. Translational Psychiatry. 2015; 5: e491 10.1038/tp.2014.133 25562841 PMC4312827

[13] Martinez D, Orlowska D, Narendran R, Slifstein M, Liu F, Kumar D, et al Dopamine Type 2/3 Receptor Availability in the Striatum and Social Status in Human Volunteers. Biological Psychiatry. 2010; 67: 275–278. 10.1016/j.biopsych.2009.07.037 19811777 PMC2812584

[14] Soutschek A, Burke CJ, Raja Beharelle A, Schreiber R, Weber SC, Karipidis II, et al The dopaminergic reward system underpins gender differences in social preferences. Nature Human Behaviour. 2017; 1: 819 10.1038/s41562-017-0226-y 31024122

[15] Rand DG, Brescoll VL, Everett JA, Capraro V, Barcelo H. Social heuristics and social roles: Intuition favors altruism for women but not for men. Journal of Experimental Psychology: General. 2016; 145: 389 10.1037/xge0000154 26913619

[16] Strang S, Hoeber C, Uhl O, Koletzko B, Münte TF, Lehnert H, et al Impact of nutrition on social decision making. Proceedings of the National Academy of Sciences of the United States of America. 2017; 114: 6510–6514. 10.1073/pnas.1620245114 28607064 PMC5488927

[17] Bennett JP, Piercey MF. Pramipexole—a new dopamine agonist for the treatment of Parkinson’s disease. Journal of the Neurological Sciences. 1999; 163: 25–31. 10.1016/s0022-510x(98)00307-4 10223406

[18] Pedroni A, Eisenegger C, Hartmann MN, Fischbacher U, Knoch D. Dopaminergic stimulation increases selfish behavior in the absence of punishment threat. Psychopharmacology.2014; 231: 135–141. 10.1007/s00213-013-3210-x 23900641

[19] Aydogan G, Furtner NC, Kern B, Jobst A, Müller N, Kocher MG. Oxytocin promotes altruistic punishment. Social cognitive and affective neuroscience. 2017; 12: 1740–1747. 10.1093/scan/nsx101 28981891 PMC5714236

[20] De Dreu CK, Greer LL, Handgraaf MJ, Shalvi S, Van Kleef GA, Baas M, et al The neuropeptide oxytocin regulates parochial altruism in intergroup conflict among humans. Science. 2010; 328: 1408–1411. 10.1126/science.1189047 20538951

[21] Crockett MJ, Siegel JZ, Kurth-Nelson Z, Ousdal OT, Story G, Frieband C, et al Dissociable effects of serotonin and dopamine on the valuation of harm in moral decision making. Current Biology. 2015; 25: 1852–1859. 10.1016/j.cub.2015.05.021 26144968 PMC4518463

[22] Sáez I, Zhu L, Set E, Kayser A, Hsu M. Dopamine modulates egalitarian behavior in humans. Current Biology. 2015; 25: 912–919. 10.1016/j.cub.2015.01.071 25802148 PMC4627633

[23] Ye Z, Hammer A, Camara E, Münte TF. Pramipexole modulates the neural network of reward anticipation. Human Brain Mapping. 2011; 32: 800–811. 10.1002/hbm.21067 21484950 PMC6870097

[24] Antonelli F, Ko JH, Miyasaki J, Lang AE, Houle S, Valzania F, et al Dopamine-agonists and impulsivity in Parkinson’s disease: impulsive choices vs. impulsive actions. Human Brain Mapping. 2014; 35: 2499–2506. 10.1002/hbm.22344 24038587 PMC4452224

[25] Schapira AHV, McDermott MP, Barone P, Comella CL, Albrecht S, Hsu HH, et al Pramipexole in patients with early Parkinson’s disease (PROUD): a randomised delayed-start trial. The Lancet Neurology. 2013; 12: 747–755. 10.1016/S1474-4422(13)70117-0 23726851 PMC3714436

[26] Costa A, Peppe A, Dell’Agnello G, Caltagirone C, Carlesimo GA. Dopamine and cognitive functioning in de novo subjects with Parkinson’s disease: Effects of pramipexole and pergolide on working memory. Neuropsychologia. 2009; 47: 1374–1381. 10.1016/j.neuropsychologia.2009.01.039 19428401

[27] O’Sullivan SS, Evans AH, Quinn NP, Lawrence AD, Lees AJ. Reckless generosity in Parkinson’s disease. Movement Disorders. 2010; 25: 221–223. 10.1002/mds.22687 20077470

[28] Huys QJ, Pizzagalli DA, Bogdan R, Dayan P. Mapping anhedonia onto reinforcement learning: a behavioural meta-analysis. Biology of Mood & Anxiety Disorders. 2013; 3: 12 10.1186/2045-5380-3-12 23782813 PMC3701611

[29] Colzato LS, Hommel B. Effects of estrogen on higher-order cognitive functions in unstressed human females may depend on individual variation in dopamine baseline levels. Frontiers in neuroscience. 2014; 8: 65 10.3389/fnins.2014.00065 24778605 PMC3985021

[30] Jacobs E, D'Esposito M. Estrogen shapes dopamine-dependent cognitive processes: implications for women's health. Journal of Neuroscience. 2011; 31: 5286–5293. 10.1523/JNEUROSCI.6394-10.2011 21471363 PMC3089976

[31] Ioannidis JP. Clarifications on the application and interpretation of the test for excess significance and its extensions. Journal of Mathematical Psychology. 2013; 57: 184–187. 10.1016/j.jmp.2013.03.002

[32] Button KS, Ioannidis JP, Mokrysz C, Nosek BA, Flint J, Robinson ES, et al Power failure: why small sample size undermines the reliability of neuroscience. Nature Reviews Neuroscience. 2013; 14: 365–376. 10.1038/nrn3475 23571845

